# An Opportunity to Understand Concerns about COVID-19 Vaccination: Perspectives from EMS Professionals

**DOI:** 10.3390/vaccines10030380

**Published:** 2022-03-02

**Authors:** Sarah R. MacEwan, Alice A. Gaughan, Megan E. Gregory, Laura J. Rush, Jonathan R. Powell, Jordan D. Kurth, Ashish R. Panchal, Ann Scheck McAlearney

**Affiliations:** 1 Division of General Internal Medicine, College of Medicine, The Ohio State University, Columbus, OH 43210, USA; 2 The Center for the Advancement of Team Science, Analytics, and Systems Thinking (CATALYST), College of Medicine, The Ohio State University, Columbus, OH 43210, USA; Alice.Gaughan@osumc.edu (A.A.G.); Megan.Gregory@osumc.edu (M.E.G.); Laura.Rush@osumc.edu (L.J.R.); Ann.McAlearney@osumc.edu (A.S.M.); 3 Department of Biomedical Informatics, College of Medicine, The Ohio State University, Columbus, OH 43210, USA; 4 National Registry of Emergency Medical Technicians, Columbus, OH 43229, USA; jpowell@nremt.org (J.R.P.); jkurth@nremt.org (J.D.K.); Ashish.Panchal@osumc.edu (A.R.P.); 5 Division of Epidemiology, College of Public Health, The Ohio State University, Columbus, OH 43210, USA; 6 Department of Emergency Medicine, The Ohio State University Wexner Medical Center, Columbus, OH 43210, USA; 7 Department of Family and Community Medicine, College of Medicine, The Ohio State University, Columbus, OH 43210, USA

**Keywords:** COVID-19, vaccination, vaccine hesitancy, emergency medical service

## Abstract

Some healthcare professionals, including emergency medical service (EMS) professionals, remain hesitant about receiving COVID-19 vaccines. This study sought to understand EMS professionals’ perspectives regarding COVID-19 vaccination. Using open-ended comments from a national survey deployed electronically to over 19,000 EMS professionals in April of 2021, we examined perspectives about acceptance of and hesitancy toward COVID-19 vaccines. Survey comments revealed differences in perspectives between vaccinated and unvaccinated EMS professionals regarding their personal role in improving public health through COVID-19 vaccination as well as vaccine benefits and the protection conferred by vaccination. Unvaccinated individuals also expressed concerns over the research and development of the COVID-19 vaccines that led to their decision not to get vaccinated. Individuals who were vaccinated suggested ways to increase uptake of the vaccine including having healthcare professionals serve as leaders for vaccination and educating individuals about COVID-19 vaccination through credible resources. Vaccine hesitancy remains a challenge to achieving herd immunity to COVID-19 through vaccination, even among healthcare professionals. Understanding the perspectives of those who have chosen not to be vaccinated can help direct strategies to reduce confusion and concerns. The perspectives of vaccinated individuals may also be valuable in identifying opportunities to promote vaccination in the professional setting.

## 1. Introduction

Vaccination is a critical tool in the fight against COVID-19. The vaccines available in the U.S. prevent infection and lessen severe illness [1,2,3], as well as reduce transmission [4]. Despite these benefits, vaccine hesitancy leading to the refusal of COVID-19 vaccines has become a significant barrier to achieving herd immunity through vaccination [5]. Of those 18 years or older in the U.S., only 73.4% had been fully vaccinated as of January, 2022 [6].

Despite their increased exposure to COVID-19 and risk of infection, vaccine hesitancy is present in healthcare workers, with some having delayed or refused to receive a COVID-19 vaccine [7]. Due to their high risk of exposure, the CDC recommends prioritization of healthcare workers for COVID-19 vaccination [8], and mandates have been introduced as an approach to bolster vaccination in this workforce [9]. Many states and employers, however, have not implemented COVID-19 vaccine mandates due to high levels of vaccine hesitancy. Improving our understanding of vaccine hesitancy can help inform efforts to increase voluntary vaccination as this is critical during the ongoing pandemic.

Emergency medical service (EMS) professionals, such as paramedics and emergency medical technicians (EMTs), are a critical part of the healthcare infrastructure, often serving as a first point of contact for patients [10]. Vaccination of EMS professionals is important for their health, the health of their patients, and the strength of the EMS workforce. Despite the significant role of EMS professionals and the benefits of their vaccination against COVID-19, only 70% were vaccinated according to a survey conducted in April 2021 [11]. Unfortunately, studies investigating EMS perspectives about COVID-19 vaccines and their hesitancy are lacking. As part of a larger project involving a national survey of EMS professionals and their experience with COVID-19 during the pandemic, we investigated the open-ended comments provided in survey responses to better understand EMS professionals’ perspectives about COVID-19 vaccines.

## 2. Materials and Methods

This is a subset analysis of a larger study on vaccine hesitancy for which summary results have been previously published [11]. In this electronic survey of EMS professionals, vaccination status was determined by the response to the question, “Have you received a COVID-19 vaccine?” The survey asked participants why they did or did not receive the vaccine, as well as evaluate factors related to vaccine acceptance and hesitancy including perceived risk of COVID-19, confidence in the COVID-19 vaccine, and medical mistrust, using validated or adapted scales [12,13,14]. Demographic characteristics were acquired from the National EMS Certification database. Results from the analysis of quantitative variables are described elsewhere [11]. The survey also allowed participants the option to provide comments explaining their vaccination decision as well as anything else they wished to share about COVID-19 vaccines. Qualitative analysis of these open-ended comments is the focus of this subset analysis.

The survey was distributed to a simple random sample of 19,062 EMS professionals in the National Registry of Emergency Medical Technicians’ (National Registry) database. The National Registry is the national certification agency for EMS professionals in the U.S. The database contains contact information for approximately 420,000 EMS professionals [15]. A link to the survey was emailed, with follow-up emails sent one and two weeks later [16]. The survey was distributed in April 2021, after vaccinations were widely available to these professionals in the U.S., but before full FDA approval of any COVID-19 vaccines and before COVID-19 vaccine mandates were commonplace. To evaluate non-response bias, a non-response survey was sent out to eligible participants that received an invitation to the original survey but did not submit a response. This non-response survey only asked for the participant’s vaccination status. There was not an opportunity to provide open-ended comments in the non-response survey.

Participants who did not provide open-ended comments were dropped from analysis. Descriptive statistics were computed for demographics and vaccination status. Differences in demographics between unvaccinated and vaccinated respondents were evaluated by Chi-square tests for categorical variables and independent samples *t*-test for continuous variables. Open-ended comments were coded for content using thematic analysis [17], supported by the use of the ATLAS.ti software program. All quotations are presented verbatim. This study was approved by the authors’ Institutional Review Board.

## 3. Results

A total of 2581 participants responded to the survey (response rate = 14%). Of these, 1145 (44.4%) provided at least one open-ended comment; 727 (63.5%) of those who provided comments were vaccinated, and 417 (36.4%) were unvaccinated. One participant’s vaccination status was unknown. Demographics of participants who provided at least one open-ended comment are presented in Table 1.

**Figure 1:**
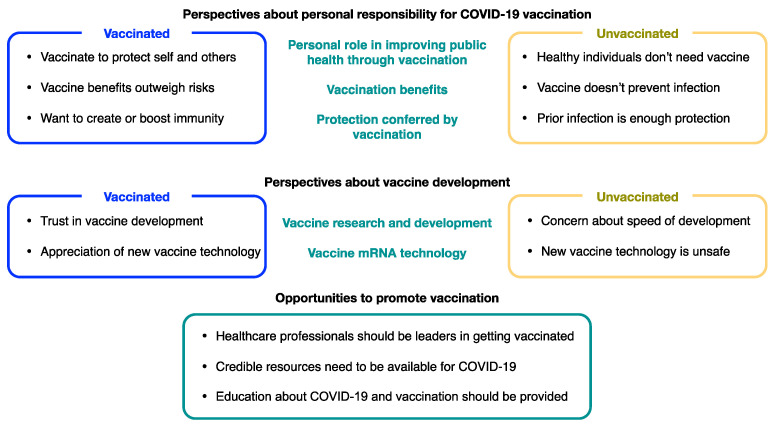
Perspectives of EMS Professionals about COVID-19 vaccination.

Open-ended responses revealed three main topics that were of particular interest regarding participants’ perspectives about COVID-19 vaccination: (1) personal responsibility for COVID-19 vaccination, (2) vaccine development, and (3) opportunities to promote vaccination. Each of these topics is discussed further below, with a summary of our findings presented in Figure 1.

### 3.1. Perspectives about Personal Responsibility for COVID-19 Vaccination

We found markedly divergent perspectives about personal responsibility for COVID-19 vaccination among vaccinated and unvaccinated participants in three areas: (1) individual role in improving public health through vaccination, (2) benefits of vaccination, and (3) protection conferred from vaccination. Contrasting perspectives of vaccinated and unvaccinated participants are presented below, with additional representative quotations provided in Table 2.

Participants were divided in their perspectives about their individual role in improving public health through vaccination. Many vaccinated respondents explained that the reason they received a COVID-19 vaccine was to protect themselves and others, whereas unvaccinated respondents rarely mentioned protecting others. One vaccinated respondent shared, “I think that everyone should get vaccinated so we can protect each other and our communities”. Unvaccinated individuals commonly commented that they did not feel there was a need for vaccination for an illness they perceived to be mild and which presented a low risk for themselves. For example, one unvaccinated respondent explained: “With such a low mortality rate and often minor illness from infection, I don’t see the need for a fit, healthy, younger person to get an experimental vaccine that is NOT certain to protect me from a disease that is still not that bad in general”.

Participants were also divided in their perspectives about COVID-19 vaccination benefits. Responses ranged from feelings that the vaccines’ benefits outweigh the risks of COVID-19 infection, to feelings that the vaccines do not work and thus have no benefit at all. For example, a vaccinated respondent explained, “The risks of COVID and side effects are far greater than the effects of receiving the vaccine. I have been vaccinated. I had some side effects, but the side effects of COVID are far worse than the vaccination”. Some unvaccinated individuals did not appreciate the role of the vaccine in reducing severe illness and doubted the benefit of a vaccine that did not completely prevent infection. One unvaccinated respondent shared, “It is not a vaccine. It is a shot that minimizes the effects of the disease. This so-called vaccine doesn’t prevent anything”.

Perspectives regarding the protection conferred by COVID-19 vaccination also differed between vaccinated and unvaccinated participants. A vaccinated respondent shared, “I chose to get a vaccine even though I had already had COVID-19. My understanding is that the immunity from the vaccination is likely to be longer lasting than that from infection”. In contrast, many unvaccinated individuals did not recognize additional protection provided by a COVID-19 vaccine if they had been previously infected. As one unvaccinated respondent noted, “If I have antibodies from getting over COVID-19, like any other, I have antibodies. I don’t need the vaccine”.

### 3.2. Perspectives about Vaccine Development

Perspectives about vaccine development were also divided. Although vaccinated participants reported trust in vaccine development, unvaccinated participants expressed concern specifically in two areas: (1) vaccine research and development, and (2) mRNA vaccine technology. Perspectives of vaccinated and unvaccinated participants about these areas are presented below, with representative quotations listed in Table 3.

Vaccinated respondents stressed that they were confident in the development of the vaccine, citing that research prior to the pandemic was valuable for vaccine development and that extraordinary research support during the pandemic allowed the vaccines to be developed quickly. One vaccinated respondent explained, “I think there are a lot of misconceptions and everyone is worried about how fast it got developed without taking into account all other research virtually stopped and that was the only thing being researched by everyone with all the funding needed”. For many unvaccinated participants, however, the vaccine research and development was a source of concern. The speed of development was one concern, as an unvaccinated respondent noted, “I do not think that this vaccine is safe due to how fast it was made and put out”. Unvaccinated participants also were concerned about a lack of information regarding evidence from the research and development process. Another unvaccinated respondent noted, “Ultimately, the reason I am not getting vaccinated is because I want to see the evidence-based research behind it all. I don’t want the first batch of it. As we saw with the Johnson and Johnson one, there are adverse effects”.

New vaccine technology was also a topic that both vaccinated and unvaccinated participants commented on, with vaccinated and unvaccinated respondents having split perspectives on mRNA vaccines. A vaccinated respondent shared, “I wish people understood that this type of vaccine, using mRNA, has been in development for much longer than the pandemic has been around”. A respondent who was unvaccinated explained they did not get the vaccine because “there is no long-term study to look at effects of mRNA vaccines on the body”.

### 3.3. Opportunities to Promote Vaccination

Vaccinated participants suggested three specific opportunities related to changing others’ perspectives about COVID-19 vaccination: (1) healthcare professionals to serve as leaders for vaccination; (2) improve the availability of credible COVID-19 vaccine-related resources; and (3) focus on efforts to educate healthcare professionals and the public on COVID-19 vaccination facts. These opportunities are discussed next, with additional representative quotations presented in Table 4.

First, comments made by vaccinated respondents highlighted that many EMS professionals felt that healthcare professionals should be leaders in obtaining vaccinations. One respondent noted, “It is our duty as EMTs to be an example to our community and show them that this is the best course of action as we know it”. Similarly, another explained the reason they received the vaccine was “to set a positive example for the community and, set the example for fellow employees”.

The need for credible resources to dispel misinformation was also highlighted as an opportunity. One respondent shared, “There is too much misinformation floating around the medical community about the SARS-CoV-2 vaccine and until this changes, it is likely that the public will remain even more misinformed”. Similarly, another noted, “There is a copious amount of misinformation about the various vaccines that even other health professionals sometimes fall victim to”.

Third, many emphasized the importance of educating healthcare professionals and the public about COVID-19 vaccines. Vaccinated individuals were frustrated with the lack of understanding of vaccines that they saw. With regard to the public, one respondent commented, “There needs to be a more concerted effort to educate the public on the facts and not the myths [about COVID-19 vaccines]”. Another stressed the need to educate professionals, such as EMS, who can then help educate the public with whom they interact: “If this vaccine is to be understood better, I think those in contact with the public need better information and education to learn and to be able to help the public become more educated. You are missing your best resource”.

## 4. Discussion

Our study revealed differing views between vaccinated and unvaccinated EMS professionals regarding the necessity and benefits of COVID-19 vaccines. These contrasting perspectives also shed light on the concerns that may contribute to vaccine hesitancy, including distrust in rapid vaccine development and uncertainty about the safety of new vaccine technology. Although our study focused on the perspectives of EMS professionals, these sentiments are reflected in other populations including other healthcare professionals [7,18] and the general public [19]. When viewed through the lens of the World Health Organization’s 3-C model of vaccine hesitancy, which considers factors of vaccine hesitancy related to confidence, complacency, and convenience [20], it was interesting to note that study participants did not express concerns related to vaccine convenience, such as vaccine availability or accessibility. This may be related to the prioritization of vaccine access for this population due to their occupational risk. Study participants did, however, express concerns related both to vaccine confidence (e.g., trust in vaccine effectiveness and safety) and complacency (e.g., lack of perceived risk of COVID-19 or benefit of vaccination), suggesting strategies to address vaccine hesitancy in populations such as first responders should focus on these factors as they impact vaccination decision making.

Participants also noted opportunities to increase vaccine acceptance, including the opportunity to lead by example, the opportunity to present credible sources of information, and the opportunity to improve education around COVID-19 and vaccination. EMS professionals and first responders were among the first groups to receive access to COVID-19 vaccines, and many were eager to be vaccinated, to both protect themselves and show their support of vaccination efforts [21,22]. Supporting this enthusiasm, we found vaccinated respondents who highlighted their opportunity to lead by example, both for their coworkers and for the public. In practice, when interacting with the public, EMS professionals can be a valuable source of information about vaccination and can relay their confidence in the vaccines. These messages can be powerful, as EMS professionals are often viewed as trusted providers in their communities [23]. Among coworkers, leading by example creates the opportunity to dispel misinformation about vaccination, while building camaraderie through commonalities in goals to conserve life, alleviate suffering, promote health, and do no harm [24]. Leadership support, open communication, and opportunities to ask questions can also help increase confidence among those individuals who have concerns that fuel their vaccine hesitancy [25,26].

Our vaccinated participants also appeared to recognize the need for credible sources of information regarding COVID-19 and COVID-19 vaccines. This need was supported by the corresponding mistrust that unvaccinated participants relayed in their comments. Many unvaccinated participants indicated that they did not trust the vaccine, reporting feeling that it was unsafe, rushed, and ineffective or not needed. These opinions were often counter to scientific evidence and in opposition to official messaging from healthcare and government sources (e.g., “The side effects of the vaccine far exceed the severity of COVID in 99% of patients”, which is directly counter to evidence showing that side effects of COVID-19 far outweigh those of the vaccine) [27].

According to a survey by the Kaiser Family Foundation, 54% of adults believe or are unsure about the validity of misinformation about the COVID-19 vaccines, including whether the vaccines can cause COVID-19, cause infertility, change DNA, contain fetal cells, or are not needed if one has already had COVID-19 [28]. These concerns were all noted by participants in our study, indicating that the pervasiveness of misinformation extends to EMS professionals. However, all of these statements are counter to scientific evidence [29]. Our prior work found that EMS professionals—particularly those who were unvaccinated against COVID-19—had low trust in the official information sources that typically communicate about this evidence, including government and healthcare sources [11]. More research is needed to understand who vaccine-hesitant individuals do trust. It is possible that trust may need to be considered on an individual level. That is, each person may look to someone they feel is in their “ingroup” for trusted information—a community member they relate to, a favored national icon, or someone who a person can simply see themselves in (e.g., similar age, race, health conditions, etc.) [30]. For the EMS population, perhaps a respected figure in the EMS profession would make an ideal vaccine role model. When considering what should be communicated by trusted individuals, the National Academies of Sciences, Engineering, and Medicine recommend that information provided be new, personally relevant, and salient in order to influence change [31].

Another challenge to be considered is addressing what happens when trust is broken. Trust violations can be considered either competence-based (i.e., unintentional, due to deficit in knowledge, mistakes) or intent-based (i.e., intentional, to advance one’s own motives at the expense of another) [32]. The former tends to be more easily repaired, whereas the latter can result in long-lasting loss of trust [32]. A common source of low trust in COVID-19 vaccines is changing information that has been commonplace throughout the pandemic [33,34]. While this was largely due to the science of COVID-19 playing out in real-time, scientifically naive individuals may have interpreted this more deviously (i.e., as an intent-based trust violation due to an ulterior motive). For example, when Dr. Anthony Fauci’s emails were released in summer 2021, showing he had told a contact in February 2020 that masking was not protective to the wearer [35], some prescribed this as a competence-based violation wherein the benefits of masking to prevent COVID-19 were not known at that early stage [36]; others positioned this as evidence of an intentional, malicious violation, such that Dr. Fauci and others were later engaged in providing purposeful false information regarding facemasks [37]. This indicates a need for messaging that recognizes information changes are related to evolving science and are to be expected. It is also important to be clear about the level of confidence in current scientific findings (e.g., we expect the vaccines will be—or can be made—effective for preventing severe disease for future variants, but it is possible a variant can develop that escapes vaccine immunity) [38], as well as about what findings are extremely unlikely to change (e.g., the COVID-19 vaccines cannot and will not change one’s DNA) [39]. This clarity may help individuals interpret changing information as a competence-based violation, which is more easily repairable in comparison to a more malicious intent-based trust violation.

Finally, vaccinated respondents recognized the need for improved education to promote vaccine acceptance. The prevalence of negative perspectives about vaccination, suggesting a need for improved understanding of vaccines, is concerning, especially among medical professionals. However, this may be less surprising among EMS professionals whose education standards do not include topics relevant to research methods, to interpretation of current vaccine literature, or to the immunological understanding of vaccines [40]. These topics are not core learning objectives in the delivery of acute lifesaving prehospital care, though they are important in other healthcare professions (e.g., physicians, nurses). Additionally, the depth of knowledge in these areas also may not be possible in the short time frames of EMS education. These educational limitations may have contributed to the susceptibility of EMS professionals to misinformation. Our findings suggest potential benefits to introducing topics relevant to vaccination, particularly related to patient and provider safety, into EMS curriculum, continuing education, or license renewal opportunities.

Sources of vaccine hesitancy found in our study are reflected in the perspectives of healthcare professionals and students in other countries [41], including similarly divided perspectives related to personal responsibility, effectiveness, and safety of booster vaccination [42]. However, it is important to note that vaccination acceptance rates in the U.S. have lagged behind other countries [43], where some countries have achieved over 90% initial vaccination as of February 2022 [44]. Notably, the U.S. also lags in booster vaccinations, achieving only 28 COVID-19 boosters per 100 people as of February 2022, in comparison to other countries where boosters have been received by greater than 50% of the population [44]. These statistics highlight the importance of addressing vaccine hesitancy in the U.S., where factors related to vaccine acceptance likely go beyond resources and access, including factors such as the politicization of vaccination that has led to pockets of unvaccinated populations [45].

Our study is limited in that it relied on the open-ended comments acquired from a larger survey that explored EMS professionals’ perspectives about COVID-19 vaccination. Response to these open-ended questions was optional, and not all survey participants provided comments. A study that focuses solely on eliciting EMS professional perspectives about the topics revealed in our analysis may provide additional depth to our understanding of the beliefs, concerns, and suggestions we found. Furthermore, our survey did have a relatively low response rate (14%); however, this response rate was typical of this study population and recruitment mechanism [46,47,48,49,50]. We did conduct a non-response survey as a part of our larger study, which demonstrated no difference in vaccination acceptance with our initial survey, supporting our belief that our low response rate did not result in biased responses [11].

## 5. Conclusions

EMS professionals have the opportunity to exemplify actions for the greater good by helping to protect the frontline workforce (e.g., first responder colleagues and hospital staff), patients, and their families and community members by being vaccinated. This study revealed beliefs and concerns about COVID-19 vaccination that may contribute to vaccine hesitancy among EMS professionals; although, these perspectives have been similarly noted in the general population. There is a heightened need to understand vaccine hesitancy and take appropriate actions to dispel it in light of the evolving information about booster shots, variants, and the reality that COVID-19 will be with us for some time. It is important to act now to reduce vaccine hesitancy among EMS professionals and the population at large.

## Figures and Tables

**Table 1:** Demographics of participants who provided at least one open-ended comment.

Characteristic	Overall	Unvaccinated (N = 417, 36.4%)	Vaccinated (N = 727, 63.5%)	*p* (Unvaccinated vs. Vaccinated)
Sex—*n* (%)				0.59
Female	377 (32.9)	141 (33.8)	236 (32.5)	
Male	753 (65.8)	269 (64.5)	483 (66.4)	
Missing	15 (1.3)	7 (1.7)	8 (1.1)	
Age—*n* (%)				<0.001
<28 years	232 (20.3)	113 (27.1)	119 (16.4)	
29–38 years	292 (25.5)	129 (30.9)	163 (22.4)	
39–50 years	296 (25.9)	93 (22.3)	202 (27.8)	
>51 years	325 (28.4)	82 (19.7)	243 (33.4)	
Race and Ethnicity—*n* (%)				0.45
White, Non-Hispanic	967 (84.5)	357 (85.6)	609 (83.8)	
All others	134 (11.7)	45 (10.8)	89 (12.2)	
Missing	44 (3.8)	15 (3.6)	29 (4.0)	
Certification—*n* (%)				0.01
Basic Life Support	418 (36.5)	172 (41.2)	246 (33.8)	
Advanced Life Support	727 (63.5)	245 (58.8)	481 (66.2)	
Educational Level—*n* (%)				<0.001
HS/GED	113 (9.9)	44 (10.6)	69 (9.5)	
Some College	327 (28.6)	146 (35.0)	181 (24.9)	
Associate’s	220 (19.2)	78 (18.7)	141 (19.4)	
Bachelor’s	250 (21.8)	69 (16.5)	181 (24.9)	
Master’s/Doctorate	79 (6.9)	18 (4.3)	61 (8.4)	
Missing	156 (13.6)	62 (14.9)	94 (12.9)	
Urbanicity—*n* (%)				0.02
Rural	390 (34.1)	167 (40.0)	222 (30.5)	
Suburban	465 (40.6)	152 (36.5)	313 (43.1)	
Urban	202 (17.6)	57 (13.7)	145 (19.9)	
Missing	88 (7.7)	41 (9.8)	47 (6.5)	
Has a health condition that makes them at high risk for increased COVID-19 disease severity—*n* (%)				<0.001
No	714 (62.4)	280 (67.1)	433 (59.6)	
Yes	346 (30.2)	99 (23.7)	247 (34.0)	
Missing	85 (7.4)	38 (9.1)	47 (6.5)	
Agency Type—*n* (%)				0.40
Fire	283 (24.7)	109 (26.1)	174 (23.9)	
Private	254 (22.2)	91 (21.8)	163 (22.4)	
Government Non-fire	149 (13.0)	53 (12.7)	95 (13.1)	
Hospital	124 (10.8)	35 (8.4)	89 (12.2)	
Other *	116 (10.1)	42 (10.1)	74 (10.2)	
Missing	219 (19.1)	87 (20.9)	132 (18.2)	
Service Type—*n* (%)				0.64
911	350 (30.6)	127 (30.5)	223 (30.7)	
All Others **	141 (12.3)	48 (11.5)	93 (12.8)	
Missing	654 (57.1)	242 (58.0)	411 (56.5)	
Years in EMS—mean (IQR)	15.1 (18.0)	12.7 (16.0)	16.5 (19.0)	<0.001
Employment Status—*n* (%)				0.22
Full-Time	666 (58.2)	247 (59.2)	418 (57.5)	
Part-Time	123 (10.7)	38 (9.1)	85 (11.7)	
Volunteer	108 (9.4)	33 (7.9)	75 (10.3)	
Missing	248 (21.7)	99 (23.7)	149 (20.5)	

Abbreviations: HS/GED, High school/General Educational Development; IQR, Interquartile range; MIHCP, Mobile Integrated Healthcare or Community Paramedicine. * Other includes air medical, tribal, military, and other; ** All Others includes medical transport, 911 and medical transport, clinical services, MIHCP, and other. *p*-values are based on Chi-square tests for categorical variables and independent samples *t*-test for continuous variables.

**Table 2:** Perspectives about personal responsibility for COVID-19 vaccination.

Topic	Verbatim Comments from Vaccinated Respondents	Verbatim Comments from Unvaccinated Respondents
Personal role in improving public health through vaccination	For my own protection and the hope that it will protect others by stopping the spread of COVID	I am a healthy, active, young woman and I do not feel I am at high risk for COVID-19 causing death.
	Wife is pregnant, got it for her and the baby’s protection	I am a healthy individual. I don’t get sick that often. I have no health issues. By personal choice I am ok letting my immune system fight it off.
	I was happy that it was provided to first responders. I am happy to have it to protect myself, my coworkers, and most important, my family at home.	I think they are great for elderly patients but the risk/reward isn’t practical for healthy adults and children.
Vaccination benefits	Benefits outweigh the risks. There is potential for side effects/adverse reactions however there is greater chance of lessening disease severity and transmission rate.	The COVID-19 vac does not and will not prevent anyone from contracting COVID and there is no evidence that it will lessen the symptoms.
	I’d rather have minimal side effects than be a COVID long hauler, or worse- dead.	From what I have seen first hand the vaccine doesn’t work and it is dangerous.
	This all boils down to risk vs. benefit. Risk-effectiveness of vaccine, long term side effects Reward-piece of mind, herd immunity.	Unsure as to the importance of receiving a vaccine for a virus for which you can still be a carrier even after the vaccine.
Protection conferred by vaccination	The immunity they [vaccines] provide is greater than naturally acquired immunity from getting infected with the virus. I was happy to get it.	If science can show my antibodies don’t last as long or are less than a vaccines, then I’ll definitely consider it.
	I contracted covid last year, as did many of my coworkers, and I still have the antibodies. But as soon as a vaccine was available, we all got it. With no history of adverse reactions from vaccinations, it was simply stupid not to.	I do not feel that those that have already had COVID-19 need to be vaccinated for it. Their body already has the antibodies.
	I already had covid. I have high antibodies but got it for further protection.	I already had COVID and survived it. My body won’t forget how to make antibodies.

**Table 3:** Perspectives about vaccine development.

Topic	Verbatim Comments from Vaccinated Respondents	Verbatim Comments from Unvaccinated Respondents
Vaccine research and development	Due to the amount of funding the pharmaceutical companies received, I believe the speed of research and development was as good as the routine R&D used by the companies under normal conditions.	I’m not willing to be a guinea pig if I don’t have to be. It was produced too quickly for my trusting.
	As a paramedic and microbiologist, I know that 30 years of research has gone into this vaccine. Without commercial investment interests governing the COVID-19 vaccine development, this went smoothly. We should do this for all vaccines moving forward.	There simply hasn’t been enough research or data to support it’s safety and effectiveness.
	I feel better knowing the basic underlying science/research began far before COVID-19.	I personally prefer to wait until I feel my family and I have more scientific research regarding the safety of these vaccines.
Vaccine mRNA technology	I am excited for the future of mRNA vaccines and medications.	MRNA is not safe, it can get into the brain barrier causing damage and possible death. There is other resources and research out there available now and people should pay attention. We’re in danger.
	While the vaccine was developed quickly, the mRNA vaccine has been around for a while. The vaccine is just as safe as any other vaccine that was developed over a longer period of time.	MRNA inoculations/modifiers have been attempted in the past and have failed long term.
	People need to know that mRNA development is much different than using any part of the virus dead/alive. Knowing that mRNA has a very short half-life and will not stay in your system for long is also huge in letting people know that it has a much smaller risk of causing any long-term effects when compared to other vaccinations.	I have specific questions about mRNA vaccines and have asked experts and gotten no answers.

**Table 4:** Opportunities to promote vaccination.

Topic	Verbatim Comments from Vaccinated Respondents
Healthcare professionals should be leaders in getting vaccinated	I feel extremely confident on the safety and protection of the vaccine. Health care professionals should be a leader on getting the vaccine.
	EMS should be setting the example. I would argue if a provider doesn’t support the science behind the vaccine, then perhaps they are in the wrong profession.
	Lead by example.
Credible resources need to be available for COVID-19	There is a lot of misinformation and disinformation out there about the vaccine and COVID-19.
	There’s too much misinformation concerning the vaccines that has been made widely available and propagated.
	I think that getting the right information out is important.
Education about COVID-19 and vaccination should be provided	I was stunned by the number of people in EMS and nursing who do not understand how vaccination technologies work and spread misinformation
	I feel that a large portion of the general public is extremely uneducated about the vaccine.
	The social stigma around vaccines shows the public has a very low amount of knowledge about science and how a mRNA vaccine works.

## Data Availability

Due to participant privacy concerns, the data presented in this study are not publicly available. The data may be requested from the corresponding author.
